# Case Report: Dual Checkpoint Inhibition in Advanced Metastatic Osteosarcoma Results in Remission of All Tumor Manifestations—A Report of a Stunning Success in a 37-Year-Old Patient

**DOI:** 10.3389/fonc.2021.684733

**Published:** 2021-08-06

**Authors:** Ulrich Sterz, Matthias Grube, Wolfgang Herr, Karin Menhart, Christina Wendl, Martin Vogelhuber

**Affiliations:** ^1^Klinik für Innere Medizin III, Universitätsklinikum Regensburg, Regensburg, Germany; ^2^Department of Nuclear Medicine, University Medical Center Regensburg, Regensburg, Germany; ^3^Zentrum für Neuroradiologie, Universitätsklinikum Regensburg, Regensburg, Germany

**Keywords:** immunotherapy, osteosarcoma, nivolumab, ipilimumab, dual checkpoint inhibition

## Abstract

**Background:**

Patients with unresectable metastasized osteosarcoma have a poor prognosis. Current treatment options do not offer a chance to cure the disease in this situation. Despite the fact that immunotherapy has expanded its indications continuously over previous years, its use is not yet established in osteosarcoma. There is a lack of randomized controlled studies that could show a significant benefit in this rare tumor entity. So far, efficacy of immunotherapy is only reported in individual cases as well as in mouse models. To predict a response to immunotherapy, testing for programmed death-ligand 1 (PD-L1) expression, microsatellite instability (MSI), and tumor mutational burden (TMB) can be useful, but status is not yet clear for most cancer entities.

**Methods:**

Single case study and review of the literature.

**Case Presentation:**

This report presents the case of a 37-year-old patient with metastatic advanced osteosarcoma, who had no more established options for tumor treatment left. PD-L1 expression in the most recent tumor sample was high (tumor proportion score (TPS) 90%, combined positive score (CPS) 92%) but no MSI could be detected. In an individual therapy attempt, an ongoing and profound remission of all tumor manifestations due to four cycles of immunotherapy with ipilimumab and nivolumab was reached. Despite discontinuation of immunotherapy for 3 months due to therapy-related pneumonitis, remission of all tumor manifestations was ongoing, and no detectable relapse in restaging before onset of Nivolumab-maintenance could be observed.

**Conclusion:**

The present case constitutes the first report of an adult patient with metastasized advanced osteosarcoma who reached a deep remission of disease by immunotherapy with ipilimumab and nivolumab, which continued even though immunotherapy had to be interrupted. To verify whether the high expression of PD-L1, as seen in this patient, is a predictive marker for response to immunotherapy in osteosarcoma, requires further investigation.

## Introduction

Osteosarcomas are rare malignant tumors of mesenchymal origin in the bone, which occur mostly in children and adolescents younger than 20 years but are also found in adults with a second peak after the age of 65 years (1). Standard treatment includes surgery framed by neoadjuvant and adjuvant chemotherapy. Commonly used agents are methotrexate (MTX), doxorubicin, cisplatin (MAP). Alternative agents are etoposide and ifosfamide (2). If all tumor manifestations can be resected, cure of the patient can be achieved, and the 5-year OS is 54% (3).

In recurrent disease, prognosis depends on the resectability of all tumor manifestations. If complete resection is also feasible in second or third relapse, cure can still be achieved. Local relapse is more favorable than recurrence in lung or other sites. Also, good histological response to pre-operative chemotherapy correlates with a more favorable outcome in recurrence (3).

However, patients with unresectable metastases have a poor prognosis, and there is no established treatment to achieve cure at this stage (4).

In advanced metastatic osteosarcoma, therapy with checkpoint inhibitors has not been established so far. In 2017, Tawbi et al. published the results of a multicenter phase 2 trial in which patients with advanced bone and soft tissue sarcomas were treated with pembrolizumab (5). Of the 22 patients with osteosarcoma, only one reached an objective response. In another multicenter phase 2 study, 85 heavily pretreated adult patients with various kinds of sarcoma received either a monotherapy with nivolumab or a combination therapy with nivolumab and ipilimumab. In the nivolumab plus ipilimumab group, six patients (16%) had a confirmed response to the immunotherapy, whereas in the nivolumab group, only two (5%) had a confirmed response (6).

A case report of a patient with advanced osteosarcoma who reached disease-control after immunotherapy with a combination of ipilimumab and nivolumab can be found in the literature (7).

Side effects of immunotherapy are frequent autoimmune phenomena of various severity, such as pneumonitis, hepatitis, or myositis (8). In the treatment of melanoma, side effects more often occur in combination therapy with CTLA-4 and PD1/PD-L1 inhibition compared with single-agent use (9). If side effects occur, discontinuation of immunotherapy and treatment with corticosteroids is required in many cases (10).

## Case Presentation

The present case reports a 37-year-old man suffering from metastatic osteosarcoma originating in the distal part of the left femur. In March 2018, the patient entered the hospital with pain in the left leg as the major symptom. An MRI scan showed a large tumor with extramedullary parts and an intraosseous diameter of 13 cm. The histological examination of the biopsy showed a mostly epithelioid, in part osteoblastic, high-grade osteosarcoma. In the CT scans of the thorax and abdomen, there was no metastasis detectable. Before surgery, the patient was treated with a neoadjuvant regimen analog to the EURAMOS-1 trial (11) with two cycles of doxorubicin and cisplatin and four cycles of high-dose MTX. In the intermediate staging performed by a further CT scan before surgical resection of the tumor, there was still no sign of distant metastasis. In the restaging-MRI of the left thigh the tumor showed a decrease in size. Limb saving surgical resection of the entire tumor (R0) was performed in August 2018. The tumor showed regression with 30% vital tumor cells (grade IV Salzer-Kutschnik).

Surgery was followed by an adjuvant chemotherapy analog to the EURAMOS-1-protocol containing two cycles of doxorubicin and cisplatin, two further cycles of Doxorubicin and eight cycles of high-dose MTX. The start of adjuvant chemotherapy was delayed for two weeks because of a wound infection.

The final staging after the last chemotherapy cycle showed two new pulmonary metastases in the CT scan of the lung. Hence, curatively intended surgical resection was performed in April 2019.

In September 2019, the patient had a seizure and in an MRI of the brain multiple cerebral metastases became visible. A neurosurgical resection of a symptomatic metastasis was performed, followed by a total brain irradiation with a boost on parafalcial and occipital metastases.

**Figure 1:**
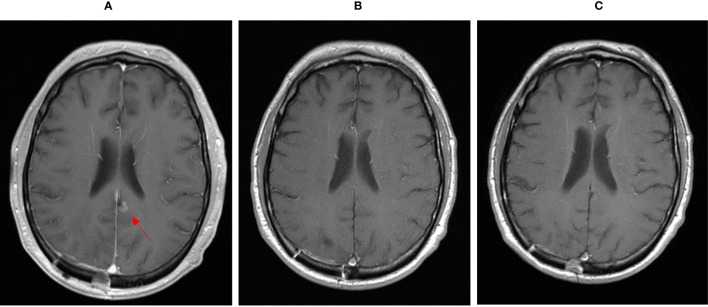
MRI showing cerebral metastasis before **(A)**, after three month **(B)** and after one year **(C)** of double check point inhibition with ipilimumab and nivolumab.

**Figure 2:**
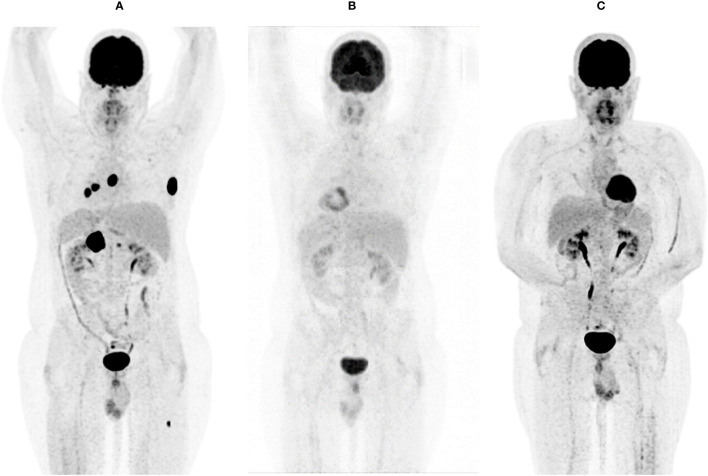
FDG-PET-CT showing systemic metastasis before **(A)**, after three months **(B)**, and after one year **(C)** of double check point inhibition with ipilimumab and nivolumab.

**Figure 3:**
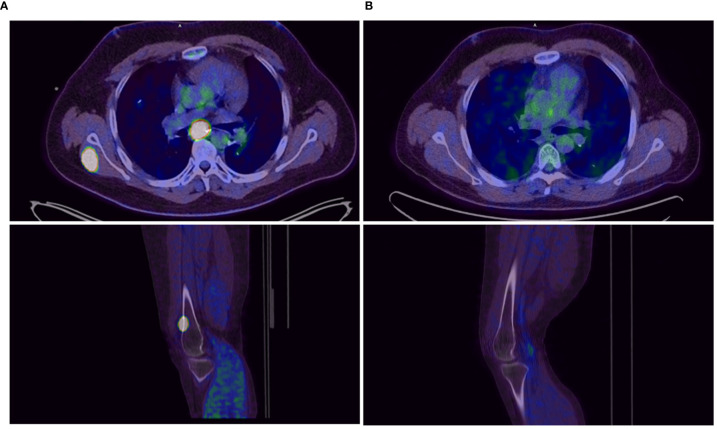
Tumor lesions before **(A)** and after 3 months **(B)** of double checkpoint inhibition with ipilimumab and nivolumab.

In a systemic restaging performed by a total body FDG-PET-CT scan and an MRI of the brain, the patient then showed a rapid systemic disease-progression with metastases affecting the lung, the mediastinum, the left adrenal gland, the brain, soft tissue, bones, and the skin. (**Figures 1A**, **2A**, **3A**)

In a molecular testing of the most recent tissue sample of the resected brain metastases, the tumor showed a high expression of PD-L1 (TPS 90% CPS 92%) but microsatellite stability (MSS). The patient was still in a good performance state (ECOG 1). A salvage chemotherapy containing the in osteosarcoma therapy established drugs ifosfamide and etoposide was not performed because of an acute kidney failure in the patient’s history and a high amount of cumulative neurotoxicity after the total brain irradiation. Benefit-risk ratio was not considered being favorable for this option. Referring to the case of a patient with advanced osteosarcoma reported by Nuytemans et al. (7), who reached a stabilization of disease-progression undergoing immunotherapy with nivolumab and ipilimumab, an individual therapy attempt with the same treatment combination was conducted, as there was no further established therapy and no ongoing study available.

Starting in December 2019, we exposed the patient to the immunotherapy combination of Nivolumab 3 mg/kg and Ipilimumab 1 mg/kg every 3 weeks for four times analog to the established treatment protocol for kidney cancer. In the following restaging performed by a PET-CT scan and an MRI of the brain 3 months after starting the therapy, the patient showed a clear response to the therapy with a profound remission of all tumor lesions (**Figures 1B**, **2B**, **3B**). In some of the lesions, a minimally elevated uptake of FDG remained residually, whereas the lesions were not metrically measurable any more in the corresponding CT scan. In brain MRIs, minimal residual structures were interpreted as gliosis after total brain irradiation and immunotherapy. A definite distinction between inflammation or scar and minimal tumor residuals was not possible in PET-CT scans and MRIs.

In February 2020, the patient suffered from herpes zoster as a complication, which was treated with brivudine for 7 days.

The patient developed a mild facial palsy of the right side in March 2020, which can be considered as a side effect of the immunotherapy. In an examination of the cerebrospinal fluid, a slightly increased cell count of 9/nl could be detected but no signs of VZV encephalitis or meningeosis carcinomatosa, respectively.

**Figure 4:**
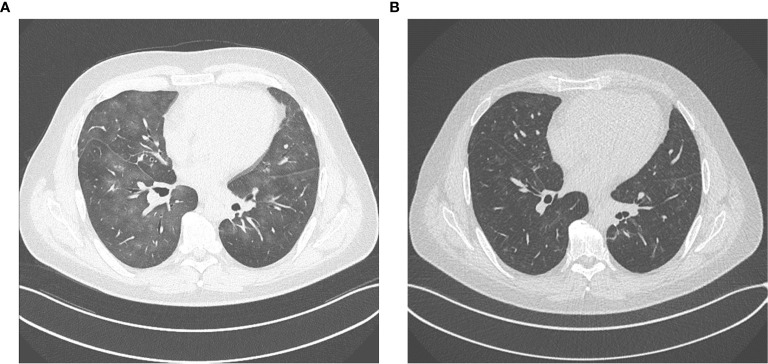
**(A)** immunotherapy related pneumonitis after four cycles of ipilimumab and nivolumab. **(B)** regression of pneumonitis after discontinuation of immunotherapy and immunosuppressive treatment with tapered prednisolone.

In March 2020, the patient developed an immunotherapy-related pneumonitis with clinically mild symptoms but clear correlations in CT scans of the lung (**Figure 4A**) and noticeably reduced diffusion capacity in a subsequent lung-function examination. Therefore, immunotherapy had to be discontinued, and nivolumab maintenance could not be started according to protocol.

For treatment of pneumonitis, the patient received prednisolone with an initial dose of 50 mg per day (0.5 mg/kg). Because of decreasing signs of pneumonitis in control CT scans (**Figure 4B**) and an improving diffusion capacity in lung function, prednisolone could be quickly tapered to 7.5 mg, and re-exposure to nivolumab was feasible in June 2020. In the actual PET-CT scan and MRI of the brain, the patient still showed a profound remission of all tumor lesions, and there was no detectable sign of a relapse (**Figures 1C**, **2C**). Currently, prednisolone is completely tapered, and the patient undergoes nivolumab maintenance (240mg) every 2 weeks. The performance state has further improved, and the patient is starting reintegration into work.

**Figure 5:**
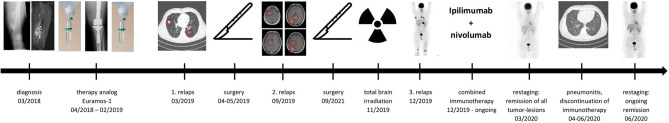
Timeline of the patients' history.

**Figure 5** outlines the patient’s history.

## Discussion

In metastatic osteosarcoma, prognosis for patients is very poor if surgical resection of all metastases is not possible (4). Standard treatment with chemotherapy often provides only little benefit with a high frequency of side effects reducing the patient’s quality of life. Radiotherapy has its place only in individual cases for local tumor control. Other treatments like therapies with different agents targeting various cellular signaling pathways are still in evaluation (12).

Approaches using immunotherapy already started in the 1970s. Although the use of interferon showed no benefit in the EURAMOS-1-trial (11), Mifamurtide showed a benefit in nonmetastatic osteosarcoma in combination with adjuvant chemotherapy (12). It is thought to be tumoricidal *via* activation of macrophages and monocytes. So far, the use of checkpoint inhibition is not an established treatment option in osteosarcoma. A phase 2 trial by Tawbi et al. treating patients with osteosarcoma with pembrolizumab showed almost no benefit (5). In addition, a pediatric phase 1/2 study testing efficacy of nivolumab in children and young adults showed no benefit in young patients with solid tumors, including osteosarcoma as well (13). A phase 1 study by Merchant, Wright et al. of Ipilimumab in pediatric patients with solid tumors, also including osteosarcoma, did not even show a partial remissions in this cohort (14). In contrast to the aforementioned results, another phase 2 trial by D’Angelo et al. in patients with mixed types of sarcomas showed a response to a combined immunotherapy with nivolumab and ipilimumab in 16% of the patients (6). Single-agent immunotherapys seems to have a limited efficacy, whereas combined immunotherapy in some patients with sarcomas leads to objective responses.

Positive predictive markers for the efficacy of immunotherapy in solid tumors, for instance PD-L1 expression in tumor and tumor environment, MSI or TMB are established, but significance is not yet clear for most entities (15). Significance of PD-L1 expression is best proven in non-small-cell lung cancer (NSCLC) and melanoma, but in a lot of other entities predictive value remains doubtful (16). In the present case, expression of PD-L1 in tumor cells (TPS), also in combination with surrounding immune cells (CPS), was high with 90% and 92%, respectively, but MSI, another positive predictive marker for success in immunotherapy in solid tumors (17, 18) was not detectable, even though it is seen in about 50% of skeletal sarcomas (19).

As a salvage chemotherapy containing ifosfamide and etoposide was expected to have an unfavorable ratio of possible benefit and harming side effects in this patient with a history of acute kidney failure and a high cumulative neurotoxicity, this option was not considered. Analog to the similar reported case, treatment with ipilimumab and nivolumab was chosen as it is established for treatment of metastasized RCC (20,) (7). For this combination, there also exists preclinical evidence concerning the efficacy against metastatic osteosarcoma in mouse model (21) and objective responses were observed in sarcoma patients (6).

Autoimmune diseases are a common side effect of immunotherapies. In double check point inhibition blocking, CTLA-4 and PD-1 severe side effects occur more frequently compared with monotherapy with a PD-1-agent (9). In the reported case the patient suffered from two probable side effects, i.e., facial palsy and pneumonitis. Standard treatment of autoimmune side effects is corticosteroids and discontinuation of immunotherapy (10), which were both done in this case. Despite of discontinuation of immunotherapy and immunosuppression with corticosteroids an ongoing anti-tumor effect was observed. Nevertheless, nivolumab maintenance was initiated after controlling side effects as in patients with melanoma resuming single-agent PD1/PD-L1-inhibition is described as feasible after toxicity in CTLA-4 and PD-L1 combination (22).

The fact that the reason for the exceptional good response to the combined immunotherapy in this patient remains unclarified constitutes the main limitation of the significance of this case report for the treatment of other patients with metastatic osteosarcoma. Hence, it currently does not seem possible to predict whether patients in similar situations would benefit from combined immunotherapy.

## Conclusion

The present case constitutes an encouraging example of a patient with advanced metastasized osteosarcoma who reached a persistent remission of all tumor lesions undergoing an immunotherapy with ipilimumab and nivolumab by analogy with the treatment protocol for advanced RCC. Immunotherapy is currently not established in osteosarcoma and efficacy of dual checkpoint inhibition in osteosarcoma has only been shown in mouse model so far (21) and has been observed in some patients with mixed types of sarcoma (6). This is the first reported case in which the patient reached a profound remission of all known tumor manifestations in metastasized osteosarcoma upon immune checkpoint inhibition. The ongoing anti-tumor-effect despite required therapy discontinuation and tapered immunosuppression with prednisolone for three months is unexpected but is indicative of a durable efficacy of immunotherapy in this patient. High PD-L1 expression of tumor cells in this case and these results of immunotherapy suggest a predictive value of PD-L1 expression but to prove this hypothesis, further research in this area is needed. The main limitation is that at the moment a predication of a response to combined immunotherapy in similar patients appears not to be possible.

## Data Availability Statement

The original contributions presented in the study are included in the article/supplementary material. Further inquiries can be directed to the corresponding author.

## Author Contributions

US and MV were responsible for treatment of the patient. KM was responsible for interpretation of PET scans. CW was responsible for interpretation of CT and MRI scans. US wrote the first draft of the manuscript. MG and WH wrote sections of the manuscript. All authors contributed to the article and approved the submitted version.

## Funding

Open access publication fees paid by Universität Regensburg.

## Conflict of Interest

The authors declare that the research was conducted in the absence of any commercial or financial relationships that could be construed as a potential conflict of interest.

## Publisher’s Note

All claims expressed in this article are solely those of the authors and do not necessarily represent those of their affiliated organizations, or those of the publisher, the editors and the reviewers. Any product that may be evaluated in this article, or claim that may be made by its manufacturer, is not guaranteed or endorsed by the publisher.

## Abbreviations

CT scan, computed tomography scan; CPS, combined positive score; CTLA-4, cytotoxic T-lymphocyte-associated Protein 4; ECOG, Eastern Cooperative Oncology Group; EURAMOS, European and American osteosarcoma study group; FDG ^18^F-Fluordesoxyglucose; MRI, magnetic resonance imaging; MSI, microsatellite instability; MSS, microsatellite stability; MTX, methotrexate; OS, overall survival; PD-L1, programmed death-ligand 1; PET, positron emission tomography; RCC, renal cell carcinoma; TMB, tumor mutational burden; TPS, tumor proportion score; VZV, varicella zoster virus.

## References

[1] Mirabello L Troisi RJ Savage SA. Osteosarcoma Incidence and Survival Rates From 1973 to 2004: Data From the Surveillance, Epidemiology, and End Results Program. Cancer (2009) 115 (7):1531–43. 10.1002/cncr.24121 PMC2813207 19197972

[2] Whelan JS Davis LE. Osteosarcoma, Chondrosarcoma, and Chordoma. J Clin Oncol (2018) 36(2):188–93. 10.1200/JCO.2017.75.1743 29220289

[3] Gelderblom H Jinks RC Sydes M Bramwell VH van Glabbeke M Grimer RJ. Survival After Recurrent Osteosarcoma: Data From 3 European Osteosarcoma Intergroup (EOI) Randomized Controlled Trials. Eur J Cancer (Oxford Engl 1990) (2011) 47(6):895–902. 10.1016/j.ejca.2010.11.036 21216138

[4] Ferrari S Briccoli A Mercuri M Bertoni F Picci P Tienghi A. Postrelapse Survival in Osteosarcoma of the Extremities: Prognostic Factors for Long-Term Survival. J Clin Oncol Off J Am Soc Clin Oncol (2003) 21(4):710–5. 10.1200/JCO.2003.03.141 12586810

[5] Tawbi HA Burgess M Bolejack V van Tine BA Schuetze SM Hu J. Pembrolizumab in Advanced Soft-Tissue Sarcoma and Bone Sarcoma (SARC028): A Multicentre, Two-Cohort, Single-Arm, Open-Label, Phase 2 Trial. Lancet Oncol (2017) 18(11):1493–501. 10.1016/S1470-2045(17)30624-1 PMC7939029 28988646

[6] D’Angelo SP Mahoney MR van Tine BA Atkins J Milhem MM Jahagirdar BN. Nivolumab With or Without Ipilimumab Treatment for Metastatic Sarcoma (Alliance A091401): Two Open-Label, Non-Comparative, Randomised, Phase 2 Trials. Lancet Oncol (2018) 19(3):416–26. 10.1016/S1470-2045(18)30006-8 PMC6126546 29370992

[7] Nuytemans L Sys G Creytens D Lapeire L. NGS-Analysis to the Rescue: Dual Checkpoint Inhibition in Metastatic Osteosarcoma - A Case Report and Review of the Literature. Acta Clin Belgica (2019) 76(2):1–6. 10.1080/17843286.2019.1683129 31635553

[8] Naidoo J Page DB Li BT Connell LC Schindler K Lacouture ME. Toxicities of the Anti-PD-1 and Anti-PD-L1 Immune Checkpoint Antibodies. Ann Oncol Off J Eur Soc Med Oncol (2015) 26(12):2375–91. 10.1093/annonc/mdv383 PMC6267867 26371282

[9] Larkin J Chiarion-Sileni V Gonzalez R Connell LC Schindler K Lacouture ME. Combined Nivolumab and Ipilimumab or Monotherapy in Untreated Melanoma. N Engl J Med (2015) 373(1):23–34. 10.1056/NEJMoa1504030 26027431 PMC5698905

[10] Puzanov I Diab A Abdallah K Bingham CO Brogdon C Dadu R. Managing Toxicities Associated With Immune Checkpoint Inhibitors: Consensus Recommendations From the Society for Immunotherapy of Cancer (Sitc) Toxicity Management Working Group. J ImmunoTher Cancer (2017) 5:95. 10.1186/s40425-017-0300-z 29162153 PMC5697162

[11] Bielack SS Smeland S Whelan JS Marina N Jovic G Hook JM. Methotrexate, Doxorubicin, and Cisplatin (Map) Plus Maintenance Pegylated Interferon Alfa-2b Versus MAP Alone in Patients With Resectable High-Grade Osteosarcoma and Good Histologic Response to Preoperative Map: First Results of the EURAMOS-1 Good Response Randomized Controlled Trial. J Clin Oncol (2015) 33(20):2279–87. 10.1200/JCO.2014.60.0734 PMC4486345 26033801

[12] Lilienthal I Herold N. Targeting Molecular Mechanisms Underlying Treatment Efficacy and Resistance in Osteosarcoma: A Review of Current and Future Strategies. Int J Mol Sci (2020) 21(18):6885. 10.3390/ijms21186885 PMC7555161 32961800

[13] Davis KL Fox E Merchant MS Marina N Jovic G Hook JM. Nivolumab in Children and Young Adults With Relapsed or Refractory Solid Tumours or Lymphoma (ADVL1412): A Multicentre, Open-Label, Single-Arm, Phase 1–2 Trial. Lancet Oncol (2020) 21(4):541–50. 10.1016/S1470-2045(20)30023-1 PMC7255545 32192573

[14] Merchant MS Wright M Baird K Wexler LH Rodriguez-Galindo C Bernstein D. Phase I Clinical Trial of Ipilimumab in Pediatric Patients With Advanced Solid Tumors. Clin Cancer Res An Off J Am Assoc Cancer Res (2016) 22(6):1364–70. 10.1158/1078-0432.CCR-15-0491 PMC5027962 26534966

[15] Shen H Yang ES-H Conry M Wexler LH Rodriguez-Galindo C Bernstein D. Predictive Biomarkers for Immune Checkpoint Blockade and Opportunities for Combination Therapies. Genes Dis (2019) 6(3):232–46. 10.1016/j.gendis.2019.06.006 PMC6997608 32042863

[16] Gibney GT Weiner LM Atkins MB. Predictive Biomarkers for Checkpoint Inhibitor-Based Immunotherapy. Lancet Oncol (2016) 17(12):e542–51. 10.1016/S1470-2045(16)30406-5 PMC5702534 27924752

[17] Le DT Uram JN Wang H Bartlett BR Kemberling H Eyring AD. Pd-1 Blockade in Tumors With Mismatch-Repair Deficiency. New Engl J Med (2015) 372(26):2509–20. 10.1056/NEJMoa1500596 PMC4481136 26028255

[18] Le DT Durham JN Smith KN Wang H Bartlett BR Aulakh LK. Mismatch Repair Deficiency Predicts Response of Solid Tumors to PD-1 Blockade. Science (2017) 357(6349):409–13. 10.1126/science.aan6733 PMC5576142 28596308

[19] Martin SS Hurt WG Hedges LK. Microsatellite Instability in Sarcomas. Ann Surg Oncol (1998) 5(4):356–60. 10.1007/BF02303500 PMC6775629 9641458

[20] Motzer RJ Tannir NM McDermott DF Arén Frontera O Melichar B Choueiri TK. Nivolumab Plus Ipilimumab Versus Sunitinib in Advanced Renal-Cell Carcinoma. N Engl J Med (2018) 378(14):1277–90. 10.1056/NEJMoa1712126 PMC5972549 29562145

[21] Lussier DM Johnson JL Hingorani P Blattman JN. Combination Immunotherapy With α-CTLA-4 and α-PD-L1 Antibody Blockade Prevents Immune Escape and Leads to Complete Control of Metastatic Osteosarcoma. J ImmunoTher Cancer (2015) 3(1):21. 10.1186/s40425-015-0067-z 25992292 PMC4437699

[22] Pollack MH Betof A Dearden H Rapazzo K Valentine I Brohl AS. Safety of Resuming anti-PD-1 in Patients With Immune-Related Adverse Events (irAEs) During Combined Anti-CTLA-4 and Anti-PD1 in Metastatic Melanoma. Ann Oncol (2018) 29(1):250–5. 10.1093/annonc/mdx642 PMC5834131 29045547

